# ATLS^® ^and damage control in spine trauma

**DOI:** 10.1186/1749-7922-4-9

**Published:** 2009-03-03

**Authors:** Oliver I Schmidt, Ralf H Gahr, Andreas Gosse, Christoph E Heyde

**Affiliations:** 1 Klinikum St. Georg gGmbH, Trauma Centre, Dept. of Trauma and Orthopaedic Surgery, Delitzscher Strasse 141, 04129 Leipzig, Germany; 2 Leipzig University, Department of Orthopaedic Surgery, Spine Unit, Liebigstrasse 20, 04103 Leipzig, Germany

## Abstract

Substantial inflammatory disturbances following major trauma have been found throughout the posttraumatic course of polytraumatized patients, which was confirmed in experimental models of trauma and in vitro settings. As a consequence, the principle of damage control surgery (DCS) has developed over the last two decades and has been successfully introduced in the treatment of severely injured patients. The aim of damage control surgery and orthopaedics (DCO) is to limit additional iatrogenic trauma in the vulnerable phase following major injury. Considering traumatic brain and acute lung injury, implants for quick stabilization like external fixators as well as decided surgical approaches with minimized potential for additional surgery-related impairment of the patient's immunologic state have been developed and used widely. It is obvious, that a similar approach should be undertaken in the case of spinal trauma in the polytraumatized patient. Yet, few data on damage control spine surgery are published to so far, controlled trials are missing and spinal injury is addressed only secondarily in the broadly used ATLS^® ^polytrauma algorithm. This article reviews the literature on spine trauma assessment and treatment in the polytrauma setting, gives hints on how to assess the spine trauma patient regarding to the ATLS^® ^protocol and recommendations on therapeutic strategies in spinal injury in the polytraumatized patient.

## Background

Polytraumatized patients often suffer from associated injuries of the spinal column following a major trauma (1^st ^hit) from direct and indirect mechanical forces that generated soft tissue-, organ injuries and fractures. The consecutive host reaction is characterized by a local and systemic expression and release of a vast array of pro-inflammatory mediators [1-4] misbalancing the immune system often resulting in a systemic inflammatory response syndrome (SIRS).

The extent of the trauma-induced first hit is the major prognostic parameter for the clinical outcome of the patient following multiple trauma. Nevertheless, secondary events including septic complications, and single or multiple organ dysfunction (MOD/MOF) like acute lung injury or acute respiratory distress syndrome (ARDS) determine the beneficial or adverse outcome of polytraumatized patients. These secondary events are often associated with surgical procedures, since increased interventional (surgery-related) antigenic load of the beforehand impaired immune system can aggravate systemic immunologic disturbances [5-16]. In fact, definitive (total care) spine surgery in polytraumatized patients, is accompanied by higher mortality rates in early vs. secondary operated patients [7].

This is where the ATLS^® ^protocol's proposition "do not further harm" comes into play and accelerates transfer of damage control surgery into damage control orthopaedics in traumatology [17-20].

This article reviews literature on spinal injury assessment and treatment principles in the polytraumatized patient and gives advice for diagnostic and therapeutic approaches with a special focus as well as ATLS^® ^and spine and damage control. The goal of treatment should be to balance necessary stabilization procedures and simultaneously limit secondary surgery-related iatrogenic trauma in search for the optimized outcome of the severely injured spine patient.

### Epidemiology of spinal injury in multiple trauma

The primary physician working on a severely injured patient should have a high suspicion for spinal trauma, since figures range from 13% to well over 30% of spinal injuries in polytraumatized patients [21-26]. In our patient population we documented spinal injury in 28% of 173 consecutive polytraumatized patients [23]. Another prospective study showed among 366 polytraumatized patients in 91% bony skeleton injury with spinal fracture found in 13% (n = 48) of all patients [27]. Of these, a third was in need for spinal stabilization. This complies with a 4% count of surgery-demanding spinal fractures in another cohort [28]. In addition, a strong association between severity of multiple injury and rate of spinal trauma has been found [29].

Injuries of the spine originate from motor vehicle accidents and incidental as well as fall from height in most cases [30-32]. The fracture locations differ substantially with a stratification of 1:4 in cervical vs. thoracolumbar spine [26]. Various studies report rates of cervical spine trauma between 2% [33] to 10% [34,35] of all polytraumatized patients.

### Initial treatment and diagnostic work up of the spine in the polytraumatized patient

The primary efforts in the initial phase are focused on life-saving procedures of the first "golden hour", which is known to be the time period in which life-threatening conditions following a major trauma can be cured by immediate therapeutic intervention [36]. For these reasons, and to capture all injuries in the mostly unconscious patients, different protocols have been developed, that allow for a structured assessment of the injured patient with consecutive time-sparing potential and beneficial outcome rates [37,38]. Of these, the ATLS^®^-protocol has the broadest distribution [39]. We do apply this algorithm in the polytrauma-management of all patients suffering from severe trauma.

### ATLS^® ^and the spine

**Figure 1:**
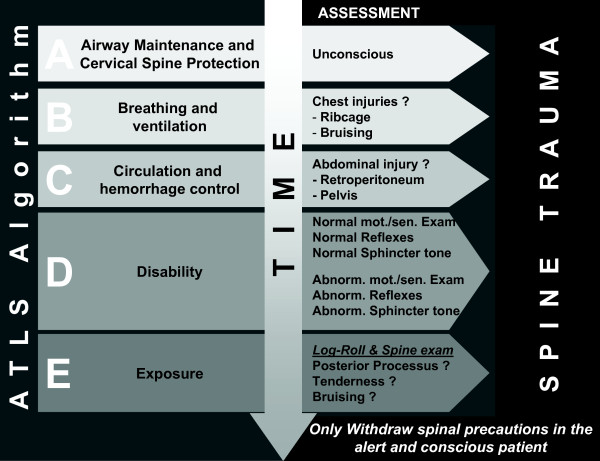
**ATLS^® ^algorithm and spine trauma assessment**. In Step „A" cervical spine (C-Spine) protection is indispensable. Every unconscious patient is stabilized by stiff-neck. Patients with signs of chest injury in step „B" and abdominal injury in step „C", especially retroperitoneal are highly suspicious for thoracic (T-) and/or (L-) lumbar spine injury. Normal motor exam and reflexes do not rule out significant spine injury in the comatose patient. Abnormal neurologic exam is a sign for substantial spinal column injury including spinal cord injury (SCI). Log roll in step „E" is important to assess the dorsum of the cervical to the sacral spine and to look out for any signs of bruising, open wounds, tender points and to palpate the paravertebral tissue and posterior processus in search for distraction injury. Spine precautions should only be discontinued when patients gain back consciousness and are alert to communicate sufficiently on spinal discomfort or neurologic sensations before the spine is cleared.

According to algorithms like the ATLS^®^-Protocol [39], interventions are focused on analyzing and restoration of a sufficient cardiopulmonary function. The algorithm in step "A" named "Airway maintenance and cervical spine protection" includes the establishment of a patent airway in association with application of a stiff-neck in the unconscious patient and the conscious patient with substantial neck pain following injury. Going through the A, B, C, D, Es a strong suspicion for spinal cord injury is entertained (see Figure 1). Specific problems arise with the patient being unconscious. Motor and sensory exam are hampered and the investigator has to rely on pathologic reflexes and weak muscle tone. Priapism and low rectal sphincter tone may count for neurological impairment e.g. paraplegia [24,25].

Since hypotension and ischemia-reperfusion are known factors for exacerbation of detrimental secondary immunologic events [2,40], the restoration of a sufficient cardiopulmonary function and consecutively constant arterial mean pressure is indispensable to maintain sufficient organ perfusion with special regard towards injuries of the central nervous system including the brain and the myelon [41,42]. This is further emphasized by the fact that immunologic secondary events following primary mechanical injury to the spinal cord and even the intervertebral disc might interact substantially with systemic immune reactions [43,44]. In consequence and according to the ATLS^® ^protocol in step B and C, early oxygenation and aggressive volume replacement is highly important [39].

The ATLS^®^-protocol also emphasizes the "log roll" in step "E" to visually inspect and manually examine the dorsal structures of the spine. The investigator can find signs of spinal trauma e.g. bruising and by palpating the processi spinosi which might be fractured or show a widened space in between, all of which counting for substantial spinal trauma [45]. As long as the patient is kept in the stiff-neck and posture is performed in axial alignment, additional injury to the spinal column is prevented and life-saving interventions (determined in the A, B, Cs) can safely be performed [31].

In addition to the clinical and radiological investigation, the event of history-taking is of significant interest regarding the injury pattern and risk for spinal injury. The physician relies on detailed information from witnesses at the scene or from the primary rescue team including the emergency doctor, paramedics and firemen. Unfortunately, handover is often insufficient and significant information is not transferred, like e.g. height of fall, level of consciousness at the injury site and fatality in the same passenger cabin [46]. Regarding spinal trauma, the event of extrication from a motor vehicle is associated with a 26 fold rate of spinal injury compared to restrained passengers [47]. Traumatic brain injury and severity of it is associated with increased risk for cervical spine trauma. Patients suffering from severe traumatic brain injury reflected by a Glasgow-Coma-Scale of 8 and below have a doubled rate of cervical spine injuries [48-51].

### Imaging of the spine in the polytrauma workup

According the original ATLS^®^-protocol, primary diagnostics include X-Ray of the pelvis, chest and a lateral view of the cervical spine [24,52,53]. If those are performed, first suspicion for thoracolumbar and cervical spine trauma can be obtained from these, like e.g. fracture of transverse process in the lower lumbar spine on the pelvis film can indicate rotational instable injury of the lumbosacral spine. For the time being, substantial argumentation about the significance of conventional **X-Ray **in the primary diagnostics exists. Some authors insist on additional anterior cervical spine and odontoid axis films to rule out around 90–95% of spinal column injuries [34]. However, under emergency room conditions and during primary survey, quality of obtained plain films is often poor. Cervicothoracal junction (C7 to T1) can hardly be imaged, especially in the obese and athletic patients with hefty soft tissues in the shoulder region. Discoligamentous injury is often not addressed by plain X-Ray [54,55]. In a recent series of 118 polytraumatized patients with cervical spine injury, in 37% of cases single lateral view failed to deliver correct diagnosis [56]. Even CT-Scan missed three patients with discoligamentous injury of the C-Spine. A similar rate of one third was found by Bohlmann somewhat 30 years ago [57]. Considering these high rates of overlooked injuries and in contrast to ATLS^® ^recommendations, even after insignificant plane x-ray the precautions should not be abandoned before the polytraumatized patient is able to communicate and give detailed information on complaints of his cervical spine [56,58,59]. Regarding thoracic and lumbar spine injuries ATLS^® ^gives no advice for diagnostic procedures in the primary survey. In brief, from our point of view, conventional X-Ray of the cervical spine does not definitely rule out cervical spine injury and should not delay primary survey in the first place. As long as stiffneck, axial posture and log-roll are performed, there is no need to enforce diagnosis of spine trauma in the primary survey of ATLS^® ^and emergency room patient workup.

With the upcoming widespread use of **CT-Scan **in the polytrauma setting, whole-body spiral scans from head to pelvis can quickly be obtained in a spiral imaging pattern. This "polytrauma" CT-Scan is performed during the secondary survey of the polytraumatized patient and many authors are in favour for a liberate indication. This we do support and suggest for every polytraumatized patient, who per definitionem has a strong suspicion for spinal trauma. High rates of initially missed spine injuries can be lowered by imaging the spine starting from C0 down to the pelvis including 2-D-Reconstruction [25,60,61]. Various reports confirm higher sensitivity and specificity of the CT-Scan versus conventional plain films in cervical spine injury [62,63]. Superposition at the cervicothoracal junction and at C0-C2, which often makes conventional x-ray useless, do not impair spatial resolution of the CT-Scan. The chance of finding additional information, like bony ligamentous avulsion or dorsal arch fractures, which might contribute to discoligamentous injury, is substantially higher in the CT-Scan [64]. This is also true for the spiral imaging acquisition in the polytrauma setting, although thickness of slices is increased to 3–5 mm compared to focused thin slice CT (1–2 mm). Image quality and various computerized reconstruction planes, e.g. sagittal and axial deliver substantial more information on the condition of the spine than any conventional plain film [65]. Regarding radiation exposure, the CT-Scan from head to pelvis generates up to threefold exposure dose than conventional plain films omitting additional specific CT-Scans to assess e.g. abdominal organ injury. For a precise classification of the fracture type additional focussed X-Ray of the injured segment is useful in some cases.

So far, **MRI **plays no role in polytrauma diagnostics [34]. This is primarily due to the fact of long exam duration and limited intervention potential during the positioning inside the apparatus [25]. In addition, regarding damage control principles, diagnostics should not delay indispensable therapeutic approaches and quick stabilization of e.g. long bone fractures is preferential to spinal trauma diagnostics.

Modern CT-Scanner with up to 32 or 64 scales are capable of obtaining a full body scan (head to pelvis) including contrast medium imaging of chest and abdominal organs in less than 3 minutes. Another fact in favour for CT-Scanning is the high rate of missed retroperitoneal injury like pancreatic or kidney lacerations as well as significant vessel injuries, all of which are often missed in the quick assessment for intraabdominal bleeding using ultrasound in the FAST^® ^protocol. The CT-Scan is undoubtedly superior concerning this matter [66-68]. The significance of CT-Scanning for polytrauma diagnostics has even resulted in installation of Scanners in the emergency room at various of the 108 level I and 209 level II trauma centres in Germany [69].

In the case of unstable hemodynamics assessed in the prehospital phase and primary survey, a different diagnostic and therapeutic approach has to be considered. If e.g. intraabdominal mass bleeding is confirmed by FAST^® ^ultrasound and immediate surgery is necessary to restore sufficient circulation, secondary survey -associated CT-Scan has to be delayed. On an individual basis the surgeon in charge has to decide whether the patient is directly transferred to the operating room. The rest of the polytrauma CT-Scan protocol should be done following emergency surgery and stabilization of the patient's condition before transfer to the ICU.

### Criteria for instability

Instability of the spinal column is defined as lack to the capability of the spinal column to prevent the myelon from injury under physiologic conditions [31]. It is imperative to obtain a precise diversification in stable and unstable spinal injury especially in the polytraumatized patient. Instable injuries of the spine should be rendered for emergent surgery according the damage control procedure, whereas stable injuries might be treated conservatively.

If plane lateral x-ray is performed or sagittal CT-Scan reconstruction is used, segmental sagittal displacement of more than 3.5 mm as well as segmental kyphosis of more than 11° might account for instability [70]. A widened intervertebral space and facet joint distraction of more than 50% might resemble instable discoligamentous injury [71]. Not specific for instable fractures is a widened prevertrebral soft tissue space. Bony avulsion injuries of the anterior or posterior upper and lower plate are seen in CT-Scan reconstructions in the first place and might point to rupture of the anterior or posterior longitudinal ligaments, which is often associated with intervertebral disc injury resulting in an instable spine. In C1, this accounts for bony avulsion injuries of the transverse ligament. Using frontal and axial reconstructions of the CT-Scan, the investigator should rule out rotational offset inside the vertebral segments, which points to instable type C fractures following axial compression or distraction in combination with rotational forces.

Nevertheless, pure discoligamentous injuries like anterior disruption through the disc (hyperextension-shear-injury, assigned type B3 according to Magerl) can sometimes not be diagnosed by a plane X-Ray or CT-Scan [56,58]. Unfortunately this is a quite frequent injury mechanism leading to instable spine injuries in e.g. headfirst pool jumpers or unrestrained car passengers. For these patients we do recommend secondary MRI imaging following initial emergency room workup and stabilization of the patients condition. If MRI is not feasible because of metallic implants like e.g. pacemaker or vessel clips, functional lateral x-rays in traction, extension and flexion or dynamic fluoroscopy can be performed by the experienced physician to visualize instability by e.g. intervertebral space widening [56,58].

In addition to these signs of instability in the cervical spine, further injuries give way for diagnosis of instable thoracic and lumbar spine trauma. Fractures, especially serial fractures of the transverse process and ribs account for instable, type C rotational injury. Patients with associated sternal fractures following hyperflexion injury in e.g. restrained motor vehicle passengers might suffer from discoligamentous posterior column injury (assigned type B) of the upper thoracic spine. In contrast, retroperitoneal bleeding as shown in contrast medium CT-Scan is often associated with instable anterior spine injury from hyperextension to the thoracolumbar region. McLain and Benson reported that anterior vertebral body height loss of more than 50%, sagittal angulation of more than 25°, three-column injury, primary neurologic deficit and serial vertebral fracture are associated with instable spine injuries [28].

### Classification and need to surgical stabilization

**Figure 2:**
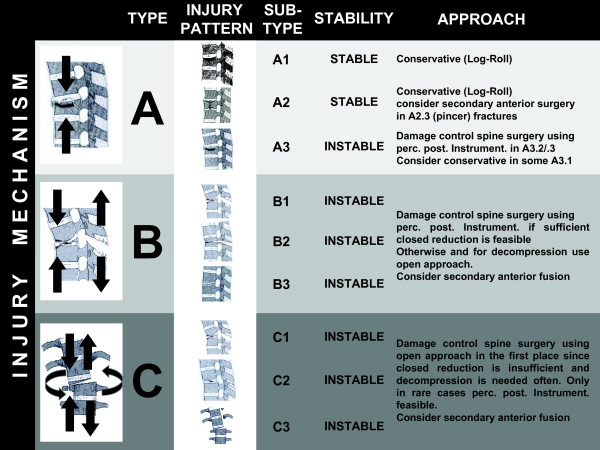
**Classification of spinal injury and treatment recommendation in the polytraumatized patient**. Classification of Magerl et al. (1993) [72] based on the two column concept of Whitesides (1977)[73]. The mechanism of applied forces to the spine generates specific fractures. Pure axial compression results in type A fractures. Distraction leads to type B and rotational momentum with compression or distraction results in type C fractures. Type A1 and A2 (except for A2.3) are regarded as stable. Whereas burst fractures, especially higher rated A3.3 lacking of sufficient anterior column support, are assigned unstable. Distraction injuries in type B1 to B3 are instable. Highest instability is seen in type C fractures with rotational moment. Conservative treatment is feasible in type A1, A2 and some lower rated A3 fractures. In these patients axial alignment and log-roll are pursued during ICU stay with subsequent mobilization and ambulation under supervision of a physiotherapist. Secondary anterior vertebral replacement might be needed in A2.3 pincer fractures. Burst fractures (A3) are characterized by their incapability to withstand anterior load that assigns them instable injuries. In A3 fractures, the high rates of overseen posterior injury should lead to liberate indication for posterior instrumentation. In B type fractures the posterior ligament complex definitely is in need of posterior instrumentation. For decompression and for insufficient reduction, open approach should be preferred, since anatomical restoration of the spinal column is the prerequisite. Rotationally instable fractures type C should be assigned to open reduction, predominantly. In addition, decompression for spinal cord injury in C-type injuries should be performed from posterior to limit second hit in polytraumatized patients. Anterior surgery in C-type fractures should be carried out in a safe period following restoration of immunologic homeostasis.

Due to a similar vertebral structure, injuries to the subaxial spinal column are classified according to Magerl et al. [72]. Various reports address this classification and the reader is kindly referred to these articles. In brief, based on the two column concept of Whitesides from 1977 [73], injuries are classified by the injuring mechanical force applied to the spine and the consecutive fracture pattern of the vertebral column (see Figure 2). Regarding the given recommendations in this section, the reader should be aware that these can only rely on a hand full of RCTs and low-quality studies that have been published so far [74-80], as well as on third opinion and the article author's personal experience. Controversial discussion regarding all questions on where, how and when to perform surgery or even use conservative treatment strategies has been going on and will endure as long as no high-quality trials are published [79,81-83], as it was brought up in a recent Cochrane review on thoracolumbar fractures [84], being able to enter only one poor-quality study into their review article which precluded firm conclusions.

### Type A fractures

Pure axial compression forces generate type A fractures. Whereas endplate fractures (type A1) and split fractures (type A2) fractures might withstand physiological axial forces and thus can be regarded stable and treated conservatively [85], vertebral burst fractures (type A3) are known for their lack of anterior support und thus are classified as instable fractures. In addition, many A3 fractures, especially type A3.3 are characterized by a substantial impairment of the spinal reserve space due to a posterior wall fragment leaking into the spinal canal. Restoration of anterior support to regain sagittal alignement of the vertebral column is generally recommended via anterior spinal surgery, e.g. corporectomy and vertebral replacement following the initial stabilization of the patient [23,26,86]. In contrast, some authors favour posterior instrumentation only [79,87] and even non-operative treatment [80], although it was shown that e.g. instrumentation without anterior column support and the intact posterior ligament complex cannot prevent posttraumatic kyphosis sufficiently, leading to posttraumatic kyphosis with potential for consecutive problems [88-91]. Regarding damage control spine surgery, the question arises, whether instable A3 fractures rendered for secondary anterior surgery should be stabilized in the trauma setting via open or minimal-invasive posterior instrumentation, first. Both respecting the biomechanical condition of the unstable spine and facilitating ICU care, spinal stabilization allows quick mobilization and prevents additional neurological impairment [92]. On the other hand, systemic effects from not stabilized spine fractures seem to be negligible when compared to long bone fractures [93].

**Figure 3:**
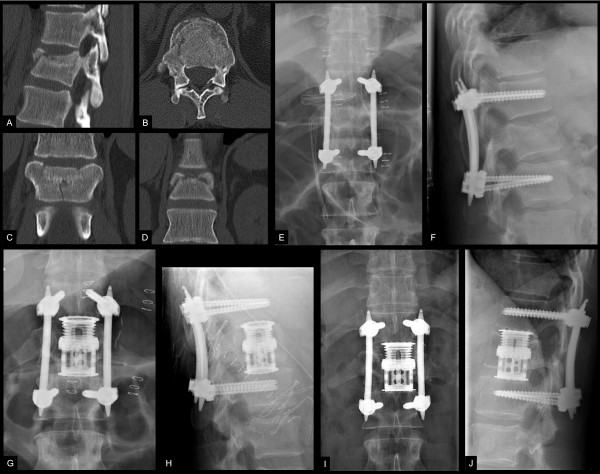
**Minimal-invasive percutaneous instrumentation and secondary anterior surgery in a polytraumatized patient with burst fracture of T12**. This is a case of a 32 year old male patient following a motor bike accident. The patient suffered from hematopneumothorax, intracapsular rupture of the liver, humeral head fracture and moderate traumatic brain injury resulting in an ISS of 34. Following primary survey and whole body CT-Scan, the patient was transferred to the OR. A chest tube was inserted and the patient was positioned prone for primary stabilization of the type A3.3 fracture of T12 (images A-D). Closed reduction and percutaneous pedicle insertion allowed quick surgery (45 minutes) and limited surgery related injury without substantial blood loss and excessive antigen load as compared to conventional open stabilization (images E-F). After uneventful recovery, definitive anterior surgery using a thoracoscopy assisted approach was performed on day 7 post trauma (images G-H). Follow-up at 24 months shows good operative result of the bisegmental fusion (images I-J).

It is evident, that in Type A fractures not seldom additional discoligamentous injuries are found, consecutively altering the classification from initial stable into unstable, which in the case of quick posterior stabilization is also addressed. If feasible, the insertion of minimal-invasive implants limits secondary hit by lesser blood loss, fast approaches and minimal soft tissue injury as reported in previous studies [94,95]. It preserves and exhibits the principles of damage control orthopaedics in spine trauma, (see Figure 3).

### Type B fractures

Distraction forces to the spinal column generate type B fractures. Posterior distraction injuries are often initially overseen or neglected, thus instable injuries are falsely regarded as stable and surgery is delayed. It is crucial to look out for signs of posterior distraction in these patients, since type B fractures are assigned unstable and require immediate stabilization in the primary operative phase [23,26,86]. To restore posterior tension banding, we use open or minimal-invasive posterior instrumentation, as mentioned beforehand.

### Type C fractures

**Figure 4:**
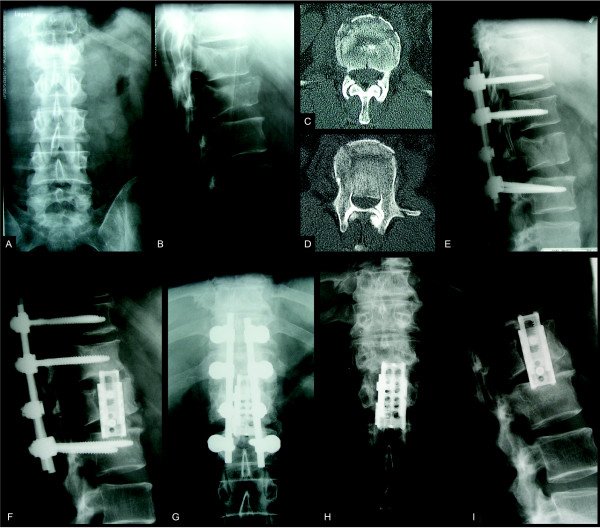
**Conventional open reduction and instrumentation with secondary anterior surgery in a polytraumatized patient with compression fracture of T12 and complete burst fracture of L1**. This case features a 39 year old male patient following a fall from height (ISS = 41). The patient was unconscious at the site of the injury and transferred after tracheal intubation to the trauma centre. Following primary survey and whole-body CT-Scan, severe traumatic brain injury with epidural hematoma, retroperitoneal bleeding with bilateral lung contusions and instable spine injuries from a complete burst fracture of L1 with substantial spinal canal compromise (type A3.3) and adjacent compression fracture of T12 (type A1.2) were revealed (images A-D). The patient was positioned prone and simultaneous surgery was performed for evacuation of epidural hematoma and stabilization of the spine. Posterior fusion using a conventional approach was performed to achieve optimized reduction of the posterior wall fragment and strongest stabilization using a cross-link and bone graft (image E). Following uneventful recovery from intracranial injuries, the patient was operated anterior using an expandable cage on day 10 post trauma (images F-G). Removal of the internal fixator after 14 months released cranial motion segment T11-T12 and showed sufficient bisegmental anterior fusion (images H-I). (Adopted from Heyde CE, Stahel PF, Ertel W. "Was gibt es Neues in der Unfallchirurgie" in: Meßmer, Jähne, Neuhaus: Was gibt es Neues in der Chirurgie? Ecomed Medizin 2005).

Axial compression or distraction forces in combination with a rotational momentum generate type C fractures. These are regarded as highly unstable and are associated with the highest rate of neurologic deficits. These fracture patterns are in need of immediate surgery, too. Although minimal-invasive percutaneous instrumentation is available, and secondary hit by limited approach related injury is favourable in the polytraumatized patient, the minimal-invasive stabilization in type C fractures plays no role, so far. Our experience is in line with others, that rotational and sagittal misalignment cannot be sufficiently addressed in a percutaneous approach in all cases [96]. In addition, cross-links to improve stability of the implanted system are not available for minimal-invasive implantation. Therefore a conventional open approach should be performed to allow for an uncompromised reduction of the spinal injury, especially in regard to eventual secondary anterior column surgery (see Figure 4). On the other hand, if sufficient reduction during posture and following traction or cautious manipulation of the patient is achieved, one should keep in mind percutaneous fixation in those rare cases [24].

### What to do with neurologic deficit in the first operative phase?

Considering spinal cord injury, a vast array of research efforts have been undertaken for we kindly refer the reader to the current literature and reviews. The consensus has been established, that a mechanical impact to the spinal cord initiates and entertains secondary injury events, that exacerbate the spinal cord injury [43,97], as it is also evident for traumatic brain injury [41,42]. As a consequence, spinal cord decompression has to be performed even in the polytraumatized patient [30] and this as quick as possible, since decompression between 24 h and 72 h is shown to be too late to prevent substantial neurologic deficits [98-102]. We therefore suggest, that following primary survey with detection of symptoms that resemble a traumatic injury to the myelon and in higher rated spinal fractures with e.g. substantial spinal canal compression from a posterior wall fragment, the extend of the operative approach has to be planned individually regarding the severity of neurological deficit, spinal fracture pattern and additional injuries with a special focus on the immunological status regarding the potential of SIRS and CARS [20]. Due to the vast array of injury combinations no guidelines can be established for a structured management of these patients. Excessive research efforts regarding pharmacological treatment options in case of neurological deficits could not show any success in clinical setting [103]. In addition, research efforts, reviews and study analyses could not confirm the results of the NASCIS-II-and NASCIS III-studies. So far, high-dosed corticosteroids have revealed no role for therapy in patients with complete traumatic spine injury and liberate indication is becoming more and more abandoned [104]. In order to not go beyond the scope of this article the interested reader is kindly referred to comprehensive articles advocating [105-108] or disclaiming [109-114] the use of Methylprednisolon.

Furthermore in incomplete paraplegia, hardly to be diagnosed in polytraumatized patients, the role of high-dosed corticosteroids remains under discussion. In respect of the before mentioned issue of secondary hit from excessive surgery in polytraumatized patients, we do suggest to favour open posterior approach including instrumentation with decompression of the spinal canal from posterior rather than anterior approach in the first operative phase.

### Damage control spine surgery

In a systematic review of retrospective studies on the timing of fracture fixation in thoracic and thoracolumbar spine trauma [115], Rutges et al. found strong support that early intervention in thoracic and lumbar spine fractures is safe and advantageous. Patients with thoracic fractures and a high ISS may benefit most from early fixation, in particular. The question arises, in which patient definitive surgery according to the principle of early total care is feasible and who is in need of a staged procedure of initial stabilization with secondary surgery. Since no data are present for the polytraumatized patient with spine injuries, one can adopt information from general orthopaedic trauma, only [36,42]. Haemodynamically instable patients with signs of shock, suffering from the lethal trias of hypothermia, coagulopathy and acidosis have highest mortality rates [116-118] and thus should be rendered for a staged procedure. In particular, a base-excess of more than – 10 mEq/l is associated with mortality rates of 40 – 70% [119,120] and elevated levels of lactate above 2 mmol/l for more than 48 hours are associated with mortality rates up to 85% [121]. Since no cut-off parameters are defined to separate into each treatment principle, the decision making has to be done on an individual basis also including associated injuries [36,116,122,123], like polytraumatized patients suffering from additional traumatic brain injury or lung contusion should be treated according to the damage control principle. Future studies should specifically address the question on where the damage control concept in spinal trauma is necessary to limit surgery related additional injury and where early total care can be performed safely.

### Secondary surgery after restoration of immunologic homoeostasis

Following initial operative stabilization of e.g. femoral fractures using external fixators and instable spine fractures using internal fixators, additional anterior surgery can be performed safely at day 7 to 10 post trauma in the uneventful recovery [2,23,30]. Conditio sine qua non is that no secondary insults e.g. infection or ARDS occurred as mentioned in the antecendent paragraphs that would prolong the hyperinflammatory status via SIRS to MODS or MOF. For instance burst fractures (Type A3) with substantial kyphotic deformation and flexion-distraction injuries (Type B) with discoligamentous injury, can be treated by e.g. anterior lateral thoracic or retroperitoneal approach without the risk of further aggravating the immunologic disturbances by the surgery-related release of pro-inflammatory mediators. This phase is generally assigned the invulnerable phase following the initial phase of hyperinflammation and secondary phase of immune paralysis. Various reports show that secondary hit from surgical approaches is best tolerated in this period around day 7 to 10 post trauma [30,124,125].

Patients suffering from prolonged SIRS or CARS are rendered for individual secondary surgery. In particular patients suffering from type C fractures of the thoracolumbar spine present with seriously elevated Injury Severity Scores (ISS) due to e.g. associated intraabdominal lacerations or lung injuries with high risk for secondary abdominal infections or ARDS, respectively. These associated injuries and complications together with the cardiopulmonary state predict the timing of secondary spine surgery in these severely injured patients. Coming from the fact that certain inflammatory mediators account for beneficial or adverse outcome in polytraumatized patients, it is without doubt, that investigators highlight immunologic monitoring as a new parameter which could be of prime importance for future planning of surgical interventions [126-128].

## Conclusion

Spinal injury in association with a polytraumatized patient is a challenge regarding diagnosis and therapeutic decision making. Precise guidelines for diagnostic workup including plane x-ray, CT-Scan and MRI do not exist, neither do therapeutic algorithms on exact timing and type of procedure, since the broad spectrum of injury patterns does not allow proposal of a structured approach or algorithm to these patients.

Nevertheless, basic recommendations for the spine trauma patient can be given. Since every polytraumatized patient should raise suspicion for serious spine injuries, it is indispensable to follow cervical as well as general spine precautions and to address potential spine trauma using the log-roll manoeuvre. To prevent adverse events considering the spine and in general, sufficient resuscitation is highly important. Diagnostics should include the use of a CT-Scanner in the first place. Conventional X-Ray remains as adjunct, only. Instable fractures should be stabilized early. The growing knowledge on the crucial role of immunologic disturbances including secondary events triggered by excessive surgery leads to a staged damage control approach. Regarding the second hit theory, excessive surgery, like anterior column reconstructions should be delayed until stable vital parameters and homeostasis are regained. The use of methylprednisolon is an option in associated incomplete spinal cord injury.

We depicted specific treatment regimes for stable and unstable fractures of the spinal column complying with a damage control approach for spine surgery in the polytraumatized patient, potentially advantageous for the patient's uneventful recovery. Future studies should address this potential, preferably in randomized-controlled trials trying to define target parameters and establish cut-off levels, as well as answering the question which patient might benefit the most.

## Consent

Written informed consent was obtained from the patient for publication of this case report and accompanying images.

A copy of the written consent is available for review by the Editor-in-Chief of this journal.

## Competing interests

RHG is member of the Spine Trauma Study Group, a non-profit organization funded by Medtronic Sofamor Danek, USA. RHG receives reimbursements for clinical evaluation of new implants from Medtronic. OIS received reimbursements for invited talks from Medtronic Sofamor Danek, USA. RHG, OIS, AG and CEH have no other financial competing interests.

## Authors' contributions

OIS and CEH were responsible for draft of the manuscript. RHG and AG reviewed the manuscript. All authors read and approved the final manuscript.
